# Variability of Visual Recovery with Time in Epiretinal Membrane Surgery: A Predictive Analysis Based on Retinal Layer OCT Thickness Changes

**DOI:** 10.3390/jcm12062107

**Published:** 2023-03-08

**Authors:** Mary Romano, Fiammetta Catania, Josè Luis Vallejo-Garcia, Tania Sorrentino, Emanuele Crincoli, Paolo Vinciguerra

**Affiliations:** 1 Multidisciplinary Department of Medical, Surgical and Dental Sciences, University of Campania “Luigi Vanvitelli”, 80138 Naples, Italy; 2 IRCCS Humanitas Research Hospital, via Manzoni 56, Rozzano, 20089 Milan, Italy; 3 Department of Biomedical Sciences, Humanitas University, Pieve Emanuele, 20090 Milan, Italy; 4 Hopital Intercommunal de Creteil, Université Paris Est Creteil, 94000 Creteil, France

**Keywords:** epiretinal membrane, optical coherence tomography, pattern, prediction, vitrectomy, visual recovery, timing

## Abstract

Purpose: To correlate postoperative optical coherence tomography (OCT) thickness changes of each retinal layer with different patterns of visual recovery after idiopathic epiretinal membrane (ERM) surgery in a cohort of patients showing no known risk factors for poor visual recovery at preoperative imaging. Methods: Best corrected visual acuity (BCVA) and OCT images were acquired preoperatively and 1 month, 3 months and 6 months postoperatively. Patients were divided into four groups according to postoperative BCVA improvement: improvement < 15 ETDRS letters (GROUP 1), immediate improvement of BCVA without further improvements at later follow-ups (GROUP 2), immediate improvement of BCVA with further improvements at later follow-ups (GROUP 3) and delayed improvement of BCVA (GROUP 4). Results: Eighty-five patients were included. GROUP1 was the only one characterized by retinal nerve fiber layer (RNFL) thickness increase and ganglion cell layer/central macular thickness (GCL/CMT) ratio decrease at 1 month and outer nuclear layer (ONL) thickness decrease at 3 and 6 months. GROUP 2 was the only one showing a decrease in GCL/inner plexiform layer (GCL/IPL) ratio at 1 month. GROUP 3 and 4 showed higher preoperative RNFL thickness compared to the other, and GROUP 4 manifested a late increase in RNFL thickness at 6 months. Conclusions: Different patterns of VA recovery are associated with specific layer thickness changes. If further confirmed, this would help detect those cases characterized by poor or delayed visual recovery despite the absence of other known imaging risk factors.

## 1. Introduction

Epiretinal membrane (ERM) is a disorder of the vitreoretinal interface with proliferation and metaplasia of cellular tissue on the surface of the retina along the inner limiting membrane (ILM) that is caused by a pathological separation of the vitreous cortex from the ILM [1,2]. ERM can be classified as idiopathic (i-ERM) or secondary to other ocular diseases, such as retinal surgery, retinal vascular disease, posterior uveitis or trauma [3]. When ERM results in a tractional effect on the retinal surface, it is a common indication for vitreoretinal surgery, because it causes not only metamorphopsia but also micropsia, macropsia, monocular diplopia and progressive vision loss [4]. The detection of ERM at an early stage has been promoted by the increasing use of optical coherence tomography (OCT) in everyday clinical practice, with a prevalence of ERM that has reached up to 34% in patients over 60 years old [5]. Vitrectomy (PPV) and ERM removal with or without ILM peeling are standard treatment procedures for management of the condition [6]. The rationale for ILM peeling stands on the fact that it serves as a scaffold for proliferation of glial cells that may migrate, creating a further increase in tangential contractile force [7]. In fact, despite the advantage in terms of functional recovery still being debated, ILM peeling has been demonstrated to be effective at reducing the risk of ERM recurrence [8,9,10,11]. Visual recovery after ERM surgery has been linked to various clinical and OCT-derived prognostic factors such as the presence of ectopic inner foveal layer (EIFL), interruption of foveal IS/OS junction and interdigitation zone, cotton wool (CW) sign and subretinal fluid (SRF), cystoid macular edema (CME), preoperative visual acuity and preoperative central macular thickness (CMT) [12]. Yet, some cases encounter a less favorable functional prognosis despite the absence of these known risk factors and independently from iatrogenic contribution. Moreover, even though it is known that the average timing of final visual acuity recovery may vary between 1 month and 6 months postoperatively, very little is known about the factors influencing the delay of this recovery. Improvements in OCT technology and automated image analysis have enabled independent measurement of each retinal layer thickness, both objectively and quantitatively [13]. Postoperative changes in retinal layer thickness have been postulated to affect visual recovery after surgery. In the present study, we aim to test the hypothesis that the variability in degree and timing of postoperative visual recovery in ERMs that do not show any preoperative or postoperative known prognostic factors might be correlated to a variability in absolute and relative postoperative thickness changes in the various foveal retinal layers.

## 2. Materials and Methods

We performed a monocentric prospective analysis of patients referred to Eye Center, Humanitas Clinical and Research Center (Rozzano, Milan, Italy) for surgical treatment of ERM between January 2017 and October 2020. All the authors reviewed the manuscript and vouched for the accuracy and completeness of the data and for the adherence of the study to the protocol (ID 1882). Informed consent and Ethics Committee approval were obtained in conformity with the Declaration of Helsinki.

**Figure 1:**
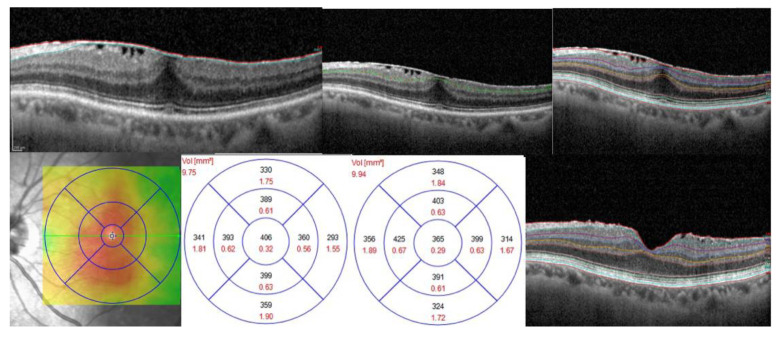
Example of retinal layer segmentation. Upper row (preoperative images): (**left**) example of bad RNFL segmentation; (**middle**) example of resegmentation of GCL; (**right**) segmentation of each retinal layer. Lower row: (**left**) preoperative heat map showing retinal thickness distribution; middle (**left**) ETDRS grid showing mean retinal thickness for each region. The central values (**fovea**) were considered for our analysis; (**middle right**) postoperative values of retinal thickness; (**Right**) postoperative segmentation.

All considered patients underwent 25-gauge pars plana vitrectomy (PPV) with ERM and ILM peeling and air tamponade. A composition of 0.125 mg Brilliant Blue G and 0.65 mg Bromphenol Blue was used for ILM and ERM staining to assist the peeling. Surgery was performed by a single expert surgeon (J.L.V.). Postoperative treatment included topical dexamethasone + levofloxacin drops 4 times per day for 1 month and topical indomethacin drops 2 times per day for 1 month. Patients underwent a baseline examination, including best corrected visual acuity (BCVA) assessment, OCT images acquisition and slit lamp examination, and were re-examined with the same procedures at 1 month, 3 months and 6 months after surgery. Inclusion criteria were pseudophakic status and preoperative diagnosis of stage II ERM [12]. ERM stage III and IV were excluded due to both inherent worse functional prognosis and poor retinal layer resolution hindering layer thickness analysis. Similarly, cases that developed surgical complications and ERMs showing other pre- or postoperative known biomarkers of negative visual prognosis (such as IS/OS interruption, foveal interdigitation zone damage, photoreceptor elongation, cotton wool sign and subretinal fluid) were excluded from the cohort. Additional exclusion criteria were choroidal neovascularization from every cause, moderate-to-severe dry age-related macular degeneration (AMD), glaucoma, high myopia (>6 diopters of refractive error), hereditary vitreoretinal diseases, history of posterior uveitis, previous vascular occlusion and diabetic retinopathy. Tomographic acquisitions were performed with Spectralis SD-OCT (Spectralis HRAþOCT, software version 5.4.7.0; Heidelberg Engineering, Inc., Heidelberg, Germany). Baseline and follow-up acquisition consisted of high-resolution images of central retinal structures with scanning dimensions of at least 20° × 15° centered on the fovea and sections ≤ 120 μm. ART parameter was set to 100. A quality score (Q) > 15 was required for analysis of the image. Automatic layer segmentation with consensus manual readjustment by two expert graders was applied to extract layer thickness data within the foveal zone. RNFL inner (anterior) boundary was considered as the outer margin of the ILM. “Inner layers” were defined as the sum of RNFL, GCL, IPL and INL, according to Spectralis segmentation program definition. The retinal thickness map analysis was then launched after verification of the correct segmentation of each layer to display numerical averages of the measurements for each of the subfields. Inner (fovea) ring (1.0 mm diameter) values were considered for the analysis. An example of how segmentation of retinal layers was performed is shown in Figure 1. According to variation in BCVA improvement after treatment, the population was further divided into 4 groups, showing either:

- No variation or improvement < 15 ETDRS letters (3 Snellen lines) [14,15] from preoperative (GROUP 1); or
- Immediate (1 month after surgery) improvement of visual acuity without further improvements at later follow-ups (GROUP 2); or
- Immediate (1 month after surgery) improvement of visual acuity with further improvements at later follow-ups (GROUP 3); or
- Delayed improvement of visual acuity (no or minimal change at 1 month follow-up and >15 ETDRS letter change at 3 or 6 months follow up) (GROUP 4).

### Statistical Analysis

Statistical analysis was conducted using SPSS software (IBM SPSS Statistics 26.0). Normality of the distribution for quantitative variables was evaluated using Shapiro–Wilk test. Normally distributed variables were described using mean and standard deviation. Qualitative variables were described as number of cases over total and percentage. One-way ANCOVA for repeated measures was used to assess variations in continuous morphological parameters in the total population. Interactions between time and VA recovery pattern in change in thickness parameters were assessed with a two-way ANCOVA for repeated measures. Multinomial logistic regression was used to detect predictors in VA recovery pattern. For all omnibus tests, post hoc analysis was performed to assess individual differences. A *p* value < 0.05 was considered as statistically significant.

## 3. Results

A total of 354 eyes were screened for inclusion. Among them, 189 were excluded due to the presence of preoperative negative prognostic biomarkers: specifically, 91 showed stage III or IV ERM; 38 showed preoperative IS/OS or interdigitation zone damage or photoreceptor elongation; 25 showed preoperative cotton wool signs or localized foveal detachment; 12 showed postoperative anatomical damage such as IS/OS interruption, interdigitation zone damage or severe dissociated optic nerve fiber layer (DONFL); and the rest (65) showed overlapping retinal disease. The selection resulted in a cohort of 85 eyes of 85 patients with a mean age of 70.73 ± 7.77 years and an almost equal sex distribution (47.0% male subjects). Chronic kidney disease, chronic obstructive pulmonary disease and systemic arterial hypertension were present in 22.4%, 23.5% and 55.3% of the patients, respectively (see Table 1).

In the total population, mean BCVA at baseline was 62.12 ± 4.9 letters and significantly improved over the course of the follow-up, with the largest relative increase registered at 1 month postoperative assessment (*p* < 0.001). Preoperative mean CMT in the total population was 483.5 ± 89.7 μm. Overall, a statistically significant reduction in both central macular thickness (CMT), inner layer thickness, retinal nerve fiber layer (RNFL), ganglion cell layer (GCL) and inner plexiform layer (IPL) thickness was observed 1 month after the treatment, also accounting for covariates (see Table 2 for detailed information about changes in each layer).

RNFL thickness was the most influenced among the above-mentioned parameters, changing from 92.57 ± 91.07 μm before surgery to 28.7 ± 25.73 μm at 1 month follow-up (*p* 0.001 at post hoc test). Nevertheless, no further significant decrease in RNFL thickness was noted after the first postoperative month. Similarly, CMT and inner layer thickness did not vary significantly after 1 month from surgery. Differently, both GCL and IPL continued to decrease at 3 months follow-up (*p* = 0.01 and *p* = 0.025, respectively, at post hoc test).

**Figure 2:**
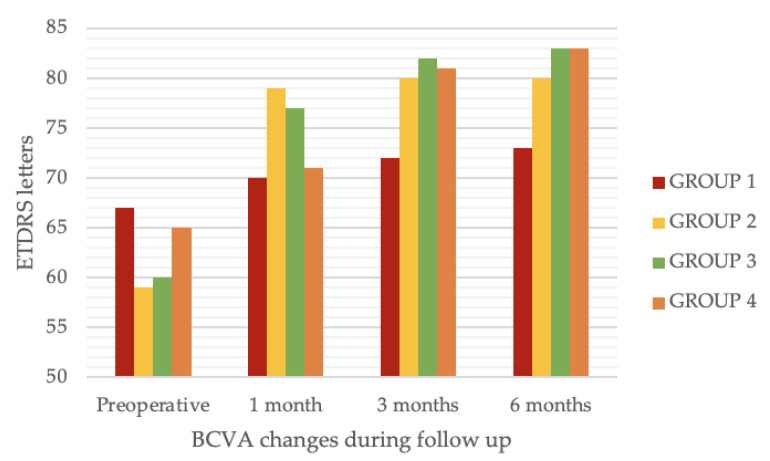
Changes in BCVA during the follow-up for each of the 4 groups. BCVA = best corrected visual acuity.

BCVA changes during the follow-up in the four study groups are shown in Figure 2.

The majority of patients treated belonged to GROUP 2 (35 patients), thus revealing immediate improvement in visual acuity without further improvements at later follow-ups as the most frequent VA recovery trend. By contrast, 15 patients experienced continuous progressive improvement (GROUP 3) and 15 patients showed a delayed recovery (GROUP 4). Lastly, 20 patients experienced insufficient VA improvement or worsening of initial visual function after treatment (GROUP 1). Table 1 describes differences in clinical and morphological parameters among the analyzed groups. A significantly higher OPL baseline thickness was present in GROUP 3 compared to all other groups (*p* = 0.078 at multinomial logistic regression). Differential thickness of RNFL, inner layers and CMT from preoperative to 3 months postoperative evaluation revealed a significantly higher difference in patients from GROUP 3 and 4 compared to the others. By contrast, analyzing differential thickness from baseline to 6-month postoperative evaluation, the CMT decrease was statistically significantly higher in GROUP 3 and lower in GROUP 1 compared to all other groups. In addition, the inner layer thickness decrease was significantly higher in GROUP 3 compared to all others.

### Distinctive OCT Thickness Variation Trends for Each Group

**Figure 3:**
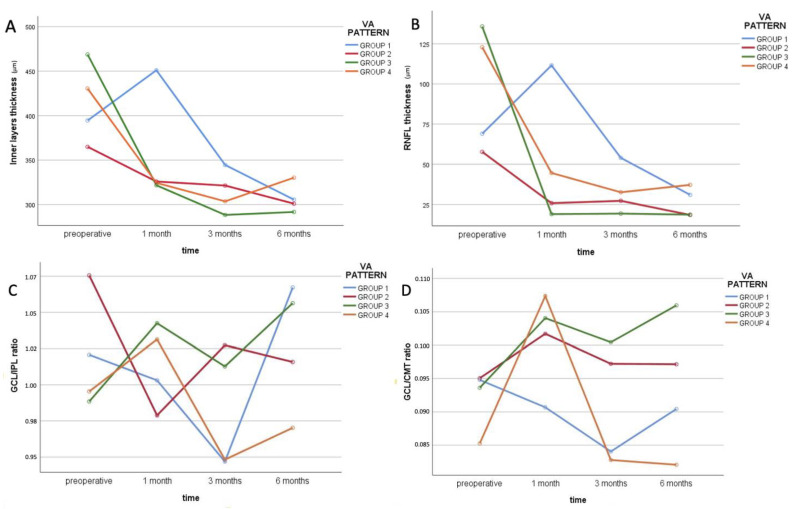
Visual representation of changes of inner layer thickness (**A**), RNFL thickness (**B**), GCL/IPL ratio (**C**) and GCL/CMT (**D**) over the follow-up in the 4 analyzed VA trend groups. CMT = central macular thickness; GCL = ganglion cell layer; IPL = inner plexiform layer; RNFL = retinal nerve fiber layer; VA = visual acuity.

Directly comparing the variation in retinal layer thickness during postoperative follow-ups in all the analyzed groups, different trends of change in inner layer, RNFL and ONL thicknesses were interestingly noted among groups (see Table 2). With regard to inner layers, GROUP 3 and GROUP 4 started from significantly higher baseline thicknesses compared to the other groups. Nevertheless, patients from GROUP 1 manifested a peculiar increase in thickness at 1 month follow-up, a behavior that was both statistically and clinically different from all others (*p* < 0.001, see Figure 3A). Analogous behavior was detected with regard to changes in RNFL thickness after treatment, with higher preoperative levels in GROUP 3 and 4 and a significant increase in thickness at 1 month follow-up in GROUP 1 differently from all others (see Figure 3B). Lastly, ONL thickness significantly decreased in GROUP 1 at 3 and 6 months follow-up compared to preoperative and 1 month follow-up values (*p* = 0.042). In all other groups, no significant change in ONL thickness was detected during follow-up.

## 4. Discussion

ERM is the most common type of fibrocellular proliferation found at the vitreoretinal interface [14,15]. Since the perfect timing and indication for surgery are still debated, it is of utmost importance to provide the most detailed prognostic information possible before and after the operation. Since the advent of OCT technology, the possibility to analyze the microscopic structure and integrity of each retinal layer has allowed us a more detailed characterization of ERMs and opened up the possibility to identify more accurate predictive parameters. Retinal thickening is a characteristic feature associated with ERM, and one would intuitively figure it to be of prognostic value, but results of such studies after ERM surgery have been inconsistent [16]. By contrast, similarly to other retinal diseases, preoperative and postoperative outer retinal layer features such as photoreceptor inner/outer segment junction and photoreceptor cone outer segment length were found to be important predictors of visual outcomes after surgery [17,18,19,20]. Nonetheless, functional surgical outcome often differs between patients independently from the status of the outer retinal layers. This has induced other authors to postulate a contribution of inner retinal layer integrity to visual recovery after surgery. Kim et al. [21] analyzed the correlation between VA recovery and thickness of each retinal layer in the foveal region of 52 eyes. They found that GCL + IPL thickness and INL thickness showed close negative association with both preoperative visual acuity and degree of metamorphopsia, while preoperative parafoveal INL thickness was strongly associated with postoperative VA. Nevertheless, they did not detect predictors of VA improvement (difference between preoperative and postoperative VA) after surgery. However, our article identified a correlation between specific trends of changes in retinal layer thickness and four different trends of VA recovery after surgery.

The first OCT thickness trend is characteristic of patients with a bad outcome (VA improvement at the end of the follow-up < 15 ETDRS letters). They manifested a significant increase in RNFL thickness at 1 month, differently from all others, and a decrease in ONL thickness at 3 and 6 months follow-up. Moreover, this group of patients was the only one characterized by a decrease in GCL/CMT ratio at 1 month follow-up (Figure 3D). We postulate that the increase in RNFL thickness in these patients might be due to RNFL swelling, which in turn could be indicative of surgically induced trauma and possible damage. This mechanism is well described in the phenomenon of the swelling of the arcuate nerve fiber layer (SANFL) after PPV and peeling of the ILM [22]. SANFL was present in 30.6% of patients in our cohort at 1 month postoperatively, which is compatible with the prevalence reported in literature [23]. Nevertheless, its presence is only marginally relevant to our analysis. In fact, not only it has been demonstrated to not influence postoperative visual acuity, but it is also typically occurring in the extrafoveal area, which was not analyzed in our investigation. Moreover, intraoperative surgical grasping is believed to be the main factor responsible for this complication. Since intraoperative grasping does not occur in the foveal region in cases of uncomplicated surgery, this causative event is to be excluded in our case. We believe that ERM peeling might induce indirect damage due to mechanical traction of the inner retinal structures occurring in the case of particularly adherent ERMs. These patients also manifested a decrease in ONL thickness after the first postoperative month. It has been demonstrated that thinning of the ONL may occur as a consequence of migration of ONL nuclei within OPL as a consequence of tractional forces exerted on the inner layers [24]. This phenomenon is made possible by the connection of ONL cell dendrites with INL cell nuclei. Taken together, the early increase in RNFL thickness and the late ONL thinning might be explained by the traumatic action of surgical traction of the inner layers in the foveal region. Coherently with this theory, postoperative VA has been negatively correlated to ERM adherence, and foveal bridging is an indicator of bad functional outcome after surgery [25,26]. Differently from Song et al. [16], we did not find a higher preoperative GCL/CMT ratio to be correlated with better visual prognosis. Nevertheless, we detected a significantly lower GCL/CMT ratio in patients from GROUP 1 compared to the other groups. Lastly, it should be reminded that the thinning of the retina in the temporal subfield and the thickening of the nasal subfield of the macula due to nasal displacement of the retina have also been reported in the literature in patients undergoing ERM surgery with ILM peeling [27]. These processes have been suggested as secondary changes resulting from axonal transport and contractility alterations in the RNFL due to apoptotic and atrophic degeneration in the peripapillary area [28].

The second trend is typical of patients manifesting immediate (1 month after surgery) improvement of VA without further improvements at later follow-ups. This course was the most prevalent in our series, which is also consistent with literature on the matter [29]. This group was the only one of the four groups showing a decrease in GCL/IPL ratio at 1 month (Figure 3C). Consistently with our results, Lee et al. [30] identified a decrease in GCL/IPL ratio as a positive prognostic factor after PPV and peeling for ERM. All the other groups from our study manifested an increase in the same parameter within the same period. The evolution of GCL/IPL over the first month may thus be considered not only as a predictor of good outcome in the short period but also as a marker of no further improvement over the remaining first year of follow-up.

The remaining two VA trends were both characterized by a delayed (after 1 month from surgery) increase in VA. One of them (GROUP 3) was the one with the best prognosis of all: these patients experienced a clinically significant VA improvement (>2 decimal lines) within the first postoperative month and continued to get better over the remaining follow-up. However, GROUP 4 showed an unsatisfactory outcome until the third postoperative month, manifesting > 2 lines of VA improvement at the end of the follow-up. Both these groups showed a significantly higher preoperative RNFL thickness compared to the other two groups. High preoperative RNFL thickness may thus be regarded at as an indicator of the possibility of late improvement in VA after surgery. We postulate that this late improvement could be attributable to the gradual relaxation of RNFL layer after the removal of longstanding traction. Unfortunately, not all patients included in the analysis were first diagnosed with ERM in our center, so we do not have reliable data on the duration of the disease before surgery. Another curious characteristic is that patients from GROUP 4 (delayed improvement) manifested a late (6 months) increase in RNFL thickness following the continuous degrading trend of the previous months. Limits of our study include the small sample size, the monocentric nature of the study, the strict inclusion and exclusion criteria and the lack of consideration for other ERM visual symptoms such as metamorphopsias. In conclusion, we propose a new OCT biomarker that could predict worse or delayed visual recovery in cases of absence of other known prognostic imaging risk factors for bad prognosis. If further confirmed, it would enhance the accuracy of preoperative and 1 month postoperative screening of patients at risk for reduced postoperative functional improvement.

## Figures and Tables

**Table 1:** Differences in anamnestic and tomographic variables between the 4 VA trend groups. Results from multinomial logistic regression are shown. BCVA = best corrected visual acuity; CKD = chronic kidney disease; CMT = central macular thickness; COPD = chronic obstructive pulmonary disease; GCL = ganglion cell layer; INL = inner nuclear layer; IPL = inner plexiform layer; OCT = optic coherence tomography; ONL = outer nuclear layer; OPL = outer plexiform layer; RNFL = retinal nerve fiber layer; SAH = systemic arterial hypertension.

Variable	Detail	GROUP 1 (20 pts)	GROUP 2 (35 pts)	GROUP 3 (15 pts)	GROUP 4 (15 pts)	*p* Regression
General data and comorbidities	Sex	8/20 (40.0%)	18/35 (51.4%)	7/15 (46.7%)	7/15 (46.7%)	0.88
	Age	72.14 ± 9.92	70.43 ± 6.88	71.57 ± 6.13	68.30 ± 9.49	0.73
	CKD	6/20 (30.0%)	6/35 (17.2%)	3/15 (20.0%)	4/15 (26.7%)	0.14
	COPD	5/20 (25.0%)	7/35 (20.0%)	4/15 (26.7%)	4/15 (26.7%)	0.26
	SAH	12/20 (60.0%)	20/35 (57.1%)	7/15 (46.7%)	8/15 (53.3%)	0.27
Visual acuity (ETDRS letters)	Preoperative BCVA	67.2 ± 4.8	59.1 ± 5.1	60.9 ± 4.9	65.1 ± 5.0	0.35
OCT preoperative macular layers (μm)	CMT	448.20 ± 87.96	464.89 ± 116.35	526.00 ± 54.19	534.29 ± 75.43	0.131
	Inner layers	355.33 ± 91.13	378.55 ± 123.60	436.88 ± 54.84	442.86 ± 84.81	0.17
	RNFL	69.93 ± 50.50	89.82 ± 106.01	108.88 ± 55.63	135.43 ± 111.31	0.44
	GCL	43.13 ± 14.31	44.31 ± 15.85	48.50 ± 15.65	44.43 ± 3.91	0.87
	IPL	42.13 ± 13.05	41.52 ± 13.07	51.00 ± 11.83	45.29 ± 5.06	0.26
	INL	55.40 ± 15.96	50.20 ± 15.69	57.38 ± 11.69	58.00 ± 13.71	0.40
	OPL	33.57 ± 9.52	36.40 ± 8.25	44.17 ± 6.71	34.43 ± 7.09	0.078
	ONL	111.21 ± 27.82	118.07 ± 30.18	134.00 ± 43.67	125.14 ± 28.73	0.47

**Table 2:** Differences in variation of retinal layer thicknesses for each follow-up according to VA trend. Results for two-way ANCOVA for repeated measures are displayed. CMT = central macular thickness; GCL = ganglion cell layer; INL = inner nuclear layer; IPL = inner plexiform layer; ONL = outer nuclear layer; OPL = outer plexiform layer; RNFL = retinal nerve fiber layer.

Variable	VA Trend	Preoperative	1 Month after Surgery	3 Months after Surgery	6 Months after Surgery	*p*	F	Partial Eta Squared
CMT	GROUP 1					0.75		
Inner layers (μm)	GROUP 1	394.50 ± 58.40	451.00 ± 44.06	344.50 ± 55.15	305.50 ± 34.70	**<0.001**	13.380	0.728
	GROUP 2	364.90 ± 24.90	325.90 ± 18.79	321.36 ± 23.51	301.00 ± 14.79			
	GROUP 3	468.66 ± 47.68	321.66 ± 35.97	288.33 ± 45.03	291.67 ± 28.33			
	GROUP 4	430.40 ± 36.94	324.40 ± 27.87	303.60 ± 34.88	330.20 ± 21.95			
RNFL (μm)	GROUP 1	69.00 ± 51.56	111.50 ± 20.69	54.00 ± 16.19	31.00 ± 14.77	**0.002**	3.85	0.418
	GROUP 2	57.73 ± 21.99	25.82 ± 8.83	27.27 ± 6.90	18.45 ± 6.29			
	GROUP 3	135.67 ± 42.10	19.00 ± 16.90	19.33 ± 13.22	18.67 ± 12.06			
	GROUP 4	122.80 ± 32.62	44.60 ± 13.09	32.60 ± 10.24	37.20 ± 9.34			
GCL (μm)						0.88		
IPL (μm)						0.37		
INL (μm)						0.55		
OPL (μm)						0.23		
ONL (μm)	GROUP 1	150.50 ± 26.41	161.50 ± 10.43	118.50 ± 14.83	120.50 ± 12.03	**0.042**	2.02	0.307
	GROUP 2	125.63 ± 11.28	121.73 ± 4.45	124.00 ± 6.32	120.00 ± 5.13			
	GROUP 3	139.33 ± 21.58	122.67 ± 8.52	108.67 ± 12.10	109.00 ± 9.82			
	GROUP 4	128.20 ± 16.72	120.00 ± 6.60	127.00 ± 9.38	131.20 ± 7.61			
GCL/IPL	GROUP 1	1.02 ± 0.19	1.00 ± 0.37	0.95 ± 0.11	1.07 ± 0.49	**0.034**	2.14	0.389
	GROUP 2	1.07 ± 0.23	0.97 ± 0.15	1.02 ± 0.18	1.02 ± 0.16			
	GROUP 3	0.99 ± 0.33	1.04 ± 0.19	1.01 ± 0.20	1.06 ± 0.13			
	GROUP 4	0.99 ± 0.16	1.03 ± 0.13	0.95 ± 0.19	0.97 ± 0.14			
GCL/CMT	GROUP 1	0.095 ± 0.012	0.090 ± 0.011	0.089 ± 0.013	0.088 ± 0.012	**0.013**	1.627	0.498
	GROUP 2	0.096 ± 0.010	0.10 ± 0.009	0.096 ± 0.011	0.097 ± 0.012			
	GROUP 3	0.093 ± 0.010	0.10 ± 0.012	0.10 ± 0.013	0.10 ± 0.010			
	GROUP 4	0.085 ± 0.012	0.10 ± 0.011	0.083 ± 0.012	0.082 ± 0.009			

Variation of retinal layer thickness during the follow-up.

## Data Availability

Data are available upon reasonable request to the corresponding author.
